# Finite Element Analysis of Von Mises Stress in External Fixators for Open Tibial Fractures: A Comparative Study of ASTM F1541‐02 and Tibia‐Based Models in Indonesian Patients

**DOI:** 10.1049/htl2.70068

**Published:** 2026-02-11

**Authors:** Muhammad Kozin, Muhammad Imam Ammarullah, Abdulfatah Abdu Yusuf, Aghni Ulma Saudi, Siti Amalina Azahra, I. Nyoman Jujur, Muhammad Hirzan Arrifqi, Moch. Agus Choiron

**Affiliations:** ^1^ Research Center for Advanced Materials Badan Riset dan Inovasi Nasional (BRIN) South Tangerang Banten Indonesia; ^2^ Bioengineering and Environmental Sustainability Research Centre University of Liberia Monrovia Montserrado Liberia; ^3^ Department of Biomedical Engineering and Health Sciences Faculty of Electrical Engineering Universiti Teknologi Malaysia Johor Bahru Johor Malaysia; ^4^ Department of Mechanical Engineering Faculty of Engineering Universitas Diponegoro Semarang Central Java Indonesia; ^5^ Department of Mechanical Engineering College of Engineering University of Liberia Monrovia Montserrado Liberia; ^6^ Zenith Allmart Precisindo Sidoarjo East Java Indonesia; ^7^ Department of Mechanical Engineering Faculty of Engineering Universitas Brawijaya Malang East Java Indonesia

**Keywords:** medical computing, biomechanics, biomedical communication, health care

## Abstract

Traffic accidents are the leading cause of traumatic fractures worldwide, with tibial fractures being the most common lower‐extremity injury. Open tibial fractures pose significant clinical challenges due to their high risk of infection and non‐union, requiring effective stabilisation. External fixators are widely used for this purpose, but their biomechanical performance must be evaluated under both standardised and patient‐specific conditions. This study presents a finite element analysis of an external fixator tailored to Indonesian patients, using two frameworks: the ASTM F1541‐02 standard protocol and a tibia‐based model derived from patient geometry. Von Mises stress distributions were assessed under axial compression, torsional loading, and four‐point bending. Results showed that stresses predicted by ASTM F1541‐02 were consistently higher than those from the tibia‐based model, particularly under torsion and bending, though all values remained below material yield strength. These findings indicate that the fixator design is safe, while emphasising that tibia‐based modelling provides more physiologically realistic predictions. The study shows the importance of patient‐specific anatomy in computational biomechanics and points to future directions in design optimisation and localized manufacturing.

## Graphical abstract

Traffic accidents are the most common cause of fractures, where tibia fractures become the most common case of lower extremity fractures. External fixators have been widely used for the treatment of open tibial fractures. The present study contributes to providing an overview of the phenomenon of external fixator installation in open tibia fracture cases for Indonesian patients.

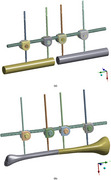

**Abbreviations**
AA 6061: Aluminum alloy 6061
ASTM: American standard testing and material
CT: Computed tomography
DICOM: Digital imaging and communications in medicine
FEM: Finite element method
IGES: Initial graphics exchange specification
MPC: Multi‐point constraint
SS 304: Stainless steel 304
SS 316L: Stainless steel 316L
Ti6Al4V: Titanium alloy

## 1 Introduction

Fractures are among the most common musculoskeletal injuries resulting from trauma [1], with traffic accidents identified as the predominant cause worldwide [2]. In Indonesia, the rising number of traffic accidents has contributed significantly to the increasing incidence of fractures. According to the Central Bureau of Statistics of the Republic of Indonesia [3], the number of traffic accidents reached 116,411 cases in 2019, reflecting an annual growth rate of 4.87%. National health data further indicate that approximately 1.3 million fracture cases occur each year in Indonesia, the highest figure in Southeast Asia. The Ministry of Health of the Republic of Indonesia reported in 2013 that 5.8% of accident victims sustained fractures, with 36.9% occurring in the upper extremities and 65.2% in the lower extremities [4]. Within the latter category, tibial fractures represent the most frequent type, accounting for 8.1–37% of lower extremity injuries annually [5].

Tibial fractures are commonly classified as open or closed [6]. Open fractures, in which a portion of the fractured bone penetrates the soft tissue and becomes exposed [7], are particularly complex to treat [8]. They are associated with high risks of infection [9], malunion [10], nonunion [11], and angular deformities [12]. Prompt and effective fixation is therefore crucial to reduce complications [13], ensure proper alignment [14], and accelerate recovery [15]. Among the various fixation methods available, external fixators have been widely recognised as a practical [16] and effective solution [17] for managing open tibia fractures. These devices realign [18] and stabilise the tibia [19] while minimising soft tissue damage [20] and reducing the risk of infection [21]. Compared with internal fixation techniques such as plates [22], screws [23], or intramedullary nails [24], external fixators provide greater stability in many cases [25] and can be adapted to different clinical conditions [26].

The development of external fixators tailored to Indonesian patients is particularly important, as the country continues to depend heavily on imported medical devices. According to the EU‐Indonesia Business Network, more than 90% of medical devices used in Indonesia are imported, creating a substantial financial burden on the national healthcare system [27]. This situation shows the urgency of developing cost‐effective external fixators that can be locally manufactured. Achieving this goal requires careful attention to several key factors. The geometry of the device must reflect the anthropometric characteristics of Indonesian patients, whose tibial dimensions are generally smaller than those of Western populations [28]. Design considerations are equally important, with uniplanar‐unilateral frames being the most widely adopted configuration in clinical practice for treating simple tibial fractures [29]. In addition, material selection plays a decisive role, and the use of accessible and affordable metals such as stainless steel 304 (SS 304) [30], aluminium alloy 6061 (AA 6061) [31], titanium alloy (Ti6Al4V) [32], and stainless steel 316L (SS 316L) [33] has been recognised as particularly promising for both clinical application [34, 35, 36, 37] and industrial feasibility [38, 39, 40, 41].

A considerable body of research on external fixators exists, spanning experimental, clinical, and computational domains. Experimental investigations, such as those reported by Su et al. [42], by Sellahewa et al. [43], and Dossanov et al. [44], have provided useful mechanical insights into device performance but are often constrained by high costs, lengthy procedures, and limited adaptability to patient‐specific geometries. Clinical studies, including those by Bangura et al. [45], Liu et al. [46], and Cao et al. [47], offer valuable evidence of practical outcomes but cannot isolate biomechanical mechanisms with sufficient precision. More recently, computational approaches based on the finite element method (FEM) have gained prominence, offering rapid [48], cost‐effective [49], and highly flexible tools [50] for evaluating biomechanical performance under various conditions. Nevertheless, most FEM‐based studies rely on a single validation framework, typically the ASTM F1541‐02 standard (as performed by Sternick et al. [51], Rosa et al. [52], and Amaro et al. [53]) or simplified tibial models (for example, Zhang et al. [54], Hemathulin et al. [55], and Guo et al. [56]), without systematic comparison of both. Moreover, these studies predominantly utilise Western anatomical datasets, which do not adequately reflect the anthropometric characteristics of Indonesian patients.

In computational biomechanics, the evaluation of stress distribution plays a central role in assessing the safety and performance of fixation devices. The von Mises stress criterion is widely applied in this context, as it provides a scalar representation of multiaxial stress states by combining the principal stress components into a single equivalent measure. This makes it particularly suitable for ductile materials such as stainless steel, titanium alloys, and aluminium alloys, which constitute the main structural components of external fixators, as conducted by Ramlee et al. [57], Mitousoudis et al. [58], and Ismail et al. [59]. Through using the von Mises formulation, regions most susceptible to yielding or mechanical failure under complex loading conditions can be identified with clarity [60]. The criterion has been consistently employed in previous finite element analyses of fixators, offering both methodological reliability and comparability across studies. Accordingly, von Mises stress was selected as the primary evaluation parameter in this study to ensure robust and clinically meaningful assessment of fixator performance.

The present study seeks to address the identified gaps by situating external fixator evaluation within the developmental trajectory of biomechanics research. Specifically, this work differs from prior investigations in three principal respects. First, it performs a direct comparative analysis of external fixators using both American Standard Testing and Material (ASTM) F 1541‐02 and tibia‐based FEM approaches within a unified computational framework, thereby clarifying the respective strengths and limitations of each method. Second, it incorporates patient‐specific geometry representative of Indonesian populations, ensuring that the results are clinically relevant to local anatomical conditions. Third, it evaluates the mechanical performance of external fixators fabricated from locally available biomaterials (SS 304, AA 6061, Ti6Al4V, and SS 316L), thereby linking computational outcomes not only to clinical safety but also to industrial feasibility and healthcare accessibility in Indonesia. Accordingly, the main objective of this study is to investigate the von Mises stress distribution in external fixators for the treatment of open tibia fractures in Indonesian patients using FEM. To this end, two computational models were developed: one based on the ASTM F 1541‐02 testing standard and the other using a tibia‐specific model derived from patient geometry. Both models were subjected to axial, torsional, and four‐point bending loads to systematically evaluate device safety and reliability. Ultimately, this research aims not only to advance scientific understanding of external fixator biomechanics but also to provide practical insights for the development of affordable, locally manufactured devices tailored to the needs of Indonesian healthcare.

## 2 Materials and Methods

### 2.1 Geometry Modelling of External Fixator

**FIGURE 1:**
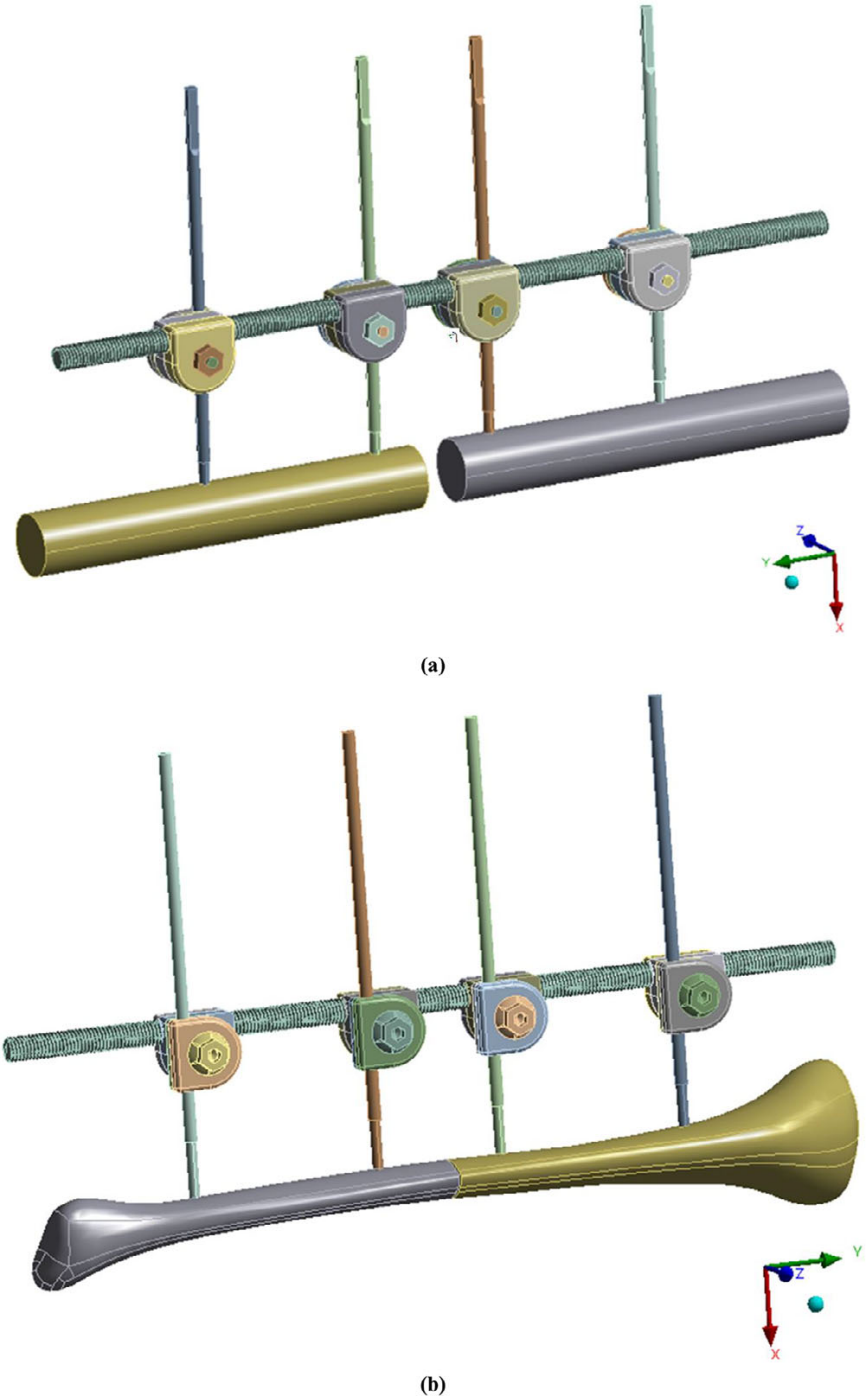
External fixator geometry modelling for different models: (a) ASTM F1541‐02 model and (b) Tibia‐based model.

The geometry of the external fixator evaluated in this study was developed under two distinct testing frameworks, namely the ASTM F 1541‐02 standard and a tibia‐based method, as illustrated in Figure 1a and b. The device modelled corresponds to a commercially available product manufactured by PT. Zenith Allmart Precisindo, Indonesia, and included its principal components: threaded bar, holder, Steinmann pin, bolt, and nut [61]. To ensure clinical relevance, the fixator design was adapted to reflect the anthropometric characteristics of Indonesian patients, whose tibial dimensions are generally smaller than those of Western populations [62]. The tibial bone model itself was provided by PT Zenith Allmart Precisindo, Indonesia, where the explained process for establishing this model was reconstructed from computed tomography (CT) scan data in digital imaging and communications in medicine (DICOM) format [63]. Image segmentation was performed using the open‐source ITK‐Snap software [64], followed by refinement of the triangulated hull surface in MeshLab [65]. The final geometry was exported in initial graphics exchange specification (IGES) format [66] and imported into the ANSYS environment for meshing and structural analysis [67].

It is important to emphasise that the tibia was modelled as an intact structure without incorporating a fracture gap or callus tissue. This simplification was deliberately chosen to maintain methodological consistency between the ASTM F1541‐02 and tibia‐based models and to isolate the effects of the testing framework on fixator stress responses. While in clinical practice open tibial fractures present with discontinuities [68] and subsequent callus formation during healing [69], replicating such conditions in silico would require extensive assumptions regarding fracture morphology [70], mechanical heterogeneity of callus tissue [71], and time‐dependent biological processes [72]. As the focus of this study was on device‐level mechanics rather than fracture healing, the simplified representation was considered appropriate. Comparable simplifications have been reported in earlier finite element studies of external fixators, including those conducted by Zhang et al. [73], Artnaseaw et al. [74], and Jung et al. [75], where bones were represented as continuous structures to enable controlled comparisons of device performance.

It should be emphasised that the present study does not replicate any specific fracture morphology, such as transverse [76], oblique [77], or comminuted [78] types. Instead, the tibia was modelled as a continuous structure to ensure identical boundary and loading conditions for both the ASTM F 1541‐02 and tibia‐based frameworks. This modelling strategy allowed the analysis to focus on the comparative mechanical performance of the external fixator without the variability introduced by different fracture configurations. In addition, soft tissue structures such as muscle [79], fat [80], and skin [81] were not incorporated into the finite element model. This exclusion was intended to reduce modelling complexity and maintain the focus on device‐level mechanics, as the inclusion of soft tissues would require numerous assumptions regarding nonlinear viscoelastic properties [82], heterogeneous material composition [83], and patient‐specific anatomical variability [84], all of which would introduce additional uncertainty. Given that the primary objective of this work was to investigate stress distribution within the external fixator rather than to replicate the full physiological environment of fracture healing, these simplifications were considered appropriate.

### 2.2 Material Properties

The material properties used in the finite element analysis for both the tibial bone and the external fixator are summarised in Tables 1 and 2, respectively [85]. The external fixator investigated in this study represents a commercial product manufactured by PT Zenith Allmart Precisindo, and its components were modelled according to their actual material compositions. Specifically, the threaded bar was modelled as SS 304, the holder as AA 6061, the Steinmann pin Ti6Al4V, and the bolts and nuts as SS 316L. These materials were selected not only to reflect the commercial configuration of the device but also because they represent widely available biomaterials in Indonesia, combining affordability, manufacturability, and clinical reliability [86, 87, 88, 89]. The key mechanical properties of these materials, including density, Poisson's ratio, Young's modulus, shear modulus, tensile strength, and yield strength, are presented in Table 3 to provide a comprehensive overview of the parameters used in the simulations.

**TABLE 1:** Material properties for tibia bone [85].

Material Property	Tibia Bone
Density(g/cm^3^)	1.4
Poisson's ratio XY (‐)	0.35
Poisson's ratio XZ (‐)	0.12
Poisson's ratio YZ (‐)	0.12
Young modulus XY (MPa)	4800
Young modulus YZ (MPa)	4800
Young modulus XZ (MPa)	9640
Shear modulus XY (MPa)	1410
Shear modulus YZ (MPa)	1280
Shear modulus XZ (MPa)	1280

**TABLE 2:** Material properties used for external fixator components [85].

Material Property	SS 304	SS 316L	Ti6Al4V	AA 6061
Density (kg/m^3^)	8000	8000	4430	2700
Poisson's ratio (‐)	0.29	0.3	0.342	0.33
Young modulus (GPa)	193	193	113.8	68.9
Shear modulus (GPa)	86	74.23	44	26
Tensile strength (MPa)	505	515	950	310
Yield strength (MPa)	215	205	880	276

**TABLE 3:** Materials used in external fixator components.

Component	Material
Threaded bar	SS 304
Holder	AA 6061
Steinman pin	Ti6Al4V
Bolt and nut	SS 316L

### 2.3 Loading Condition and Friction Coefficient

In the present study, three primary loading scenarios were investigated: axial compression, torsional loading, and four‐point bending. These conditions were applied under both the ASTM F 1541‐02 testing framework and the tibia‐based modelling framework, thereby enabling systematic comparison of external fixator performance across two commonly employed validation approaches. All simulations were conducted under static conditions with an applied load magnitude of 500 N adopted from Ali et al. [90], representing the approximate physiological force transmitted through the tibia during quiet standing in an average adult patient. This loading value has been widely adopted in previous computational and experimental biomechanical studies and provides a clinically meaningful baseline for evaluating construct stability.

To further simplify the analysis and maintain focus on device‐level stress distribution, the coefficient of friction between the external fixator components and the tibial surface was neglected, and all contacts were modelled as frictionless, which is the same as performed by Agostinis [91], Chen et al. [92], and Scott [93]. This assumption is consistent with earlier finite element investigations of external fixation systems, such as those reported by Karunratanakul et al. [94], where the primary objective was comparative mechanical assessment rather than replication of the full physiological interface. While omitting frictional effects inevitably limits the ability to capture micromotion or sliding phenomena at the bone‐pin interface, it allows for clearer interpretation of structural stress pathways within the fixator assembly and enables direct comparison between different test configurations.

The application of torsional and bending loads required particular methodological considerations. Since solid finite elements inherently lack rotational degrees of freedom, moments could not be directly imposed at the element level. Instead, torsional and bending moments were introduced using remote reference points coupled to the relevant loaded surfaces via multi‐point constraint (MPC) equations within the ANSYS environment [95]. For torsional loading, a remote point was defined at the centroid of the distal tibial cross‐section and rigidly coupled to the surface nodes, after which a torque was applied about the longitudinal axis. This approach ensured uniform transfer of rotational effects to the connected solid elements without inducing artificial stress concentrations. For four‐point bending, pure bending moments were generated by applying two equal and opposite pairs of forces at specified offsets, thereby creating a constant bending region while preventing rigid‐body displacement. These procedures follow established ANSYS practices and are consistent with techniques reported in the biomechanics literature, particularly the framework described by Roseiro et al. [96], who demonstrated their validity for orthopaedic implant simulations.

### 2.4 Finite Element Modelling of External Fixator

**FIGURE 2:**
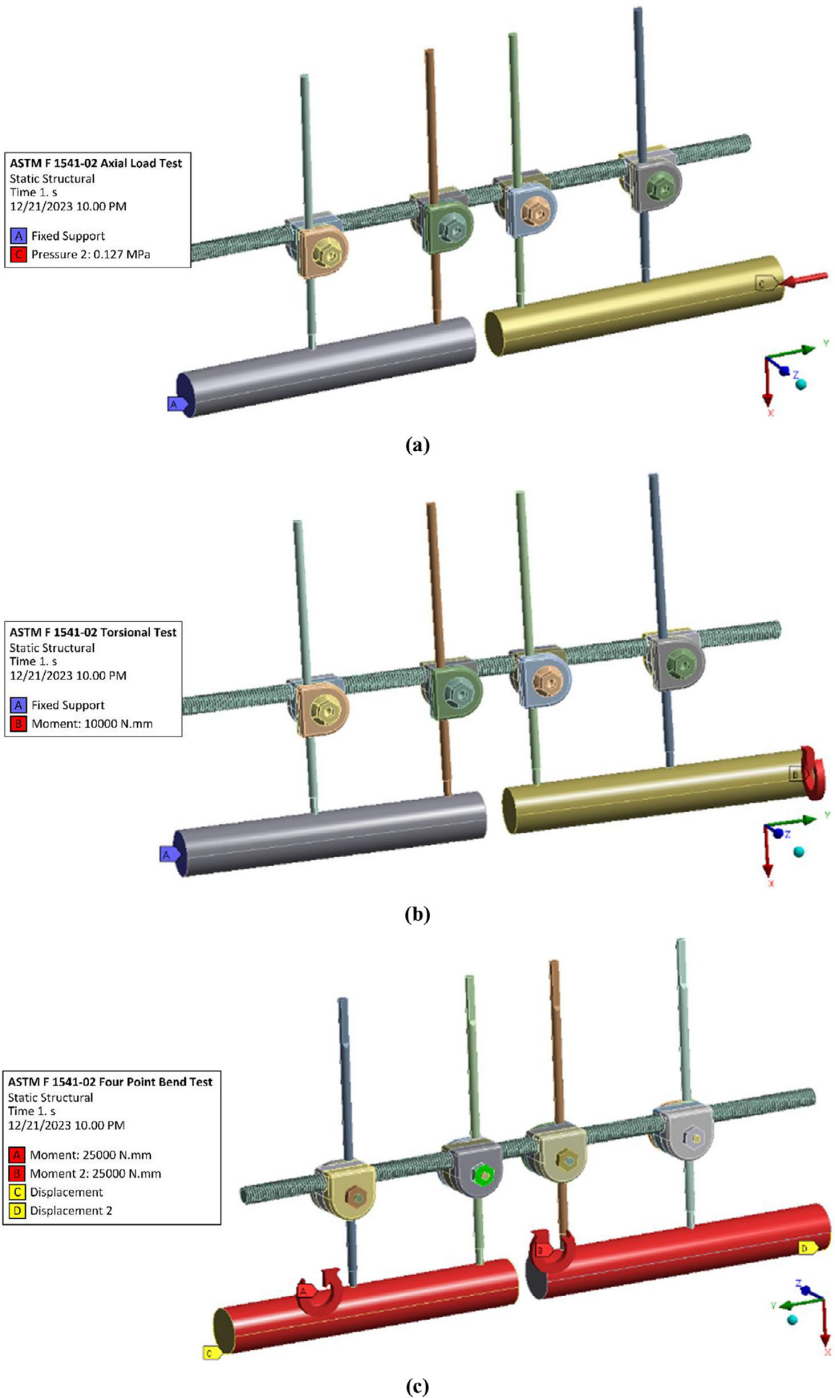
Finite element modelling of external fixator on ASTM F1541‐02 model:: (a) Axial load test, (b) Torsional test, and (c) Four‐point bend test.

**FIGURE 3:**
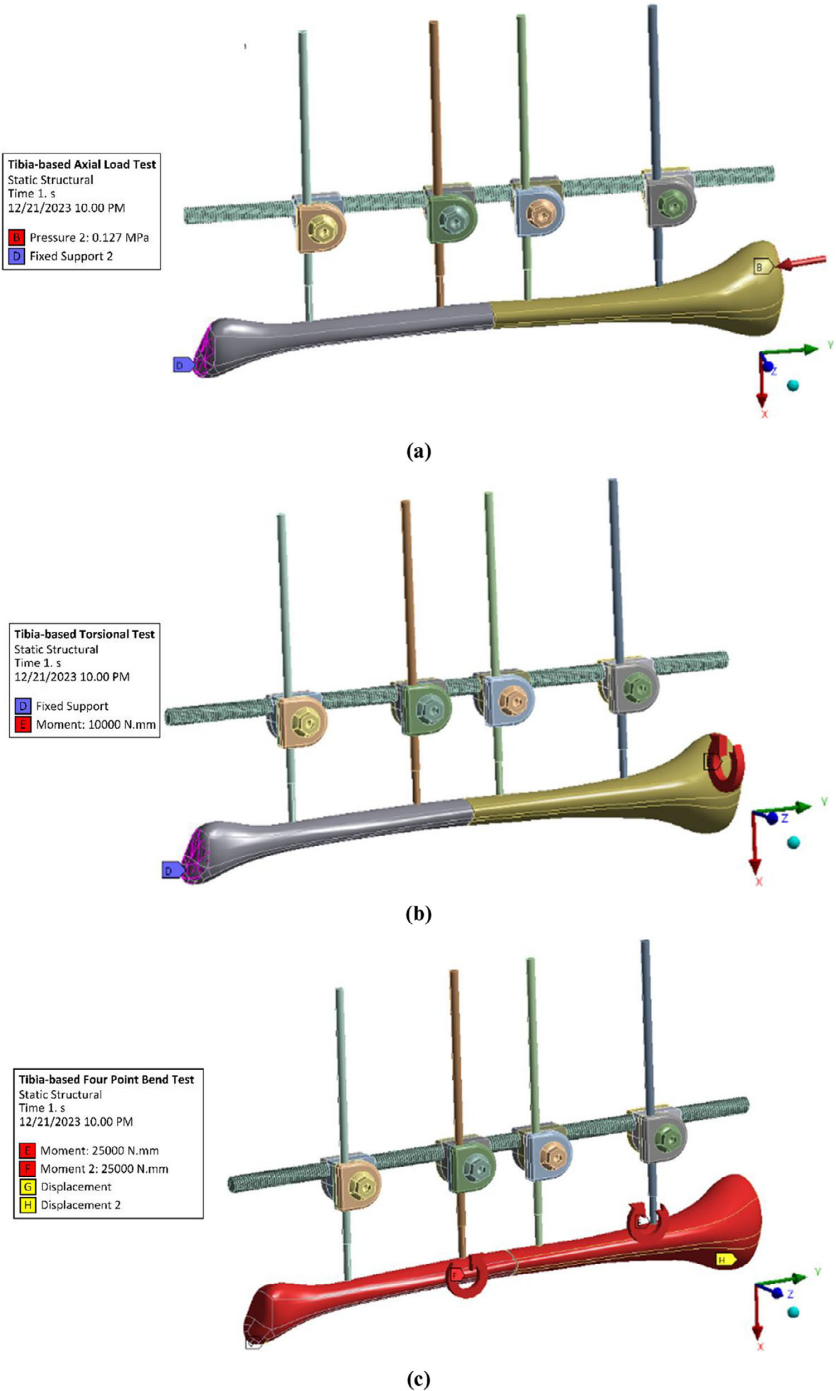
Finite element modelling of external fixator on tibia‐based model: (a) Axial load test, (b) Torsional test, and (c) Four‐point bend test.

The computational simulation of the external fixator was carried out using the FEM in ANSYS to evaluate device‐level stress distribution under clinically relevant conditions. A static structural analysis was performed in which the von Mises stress within the fixator components was quantified under three loading modes: axial compression, torsion, and four‐point bending. These loading protocols, consistent with those reported in earlier biomechanics studies such as Shi et al. [97], were applied within both the ASTM F1541‐02 testing framework and the tibia‐based framework, as illustrated in Figure 2 and Figure 3, respectively. This dual approach allowed a systematic comparison between standardised and patient‐specific models, thereby providing a broader understanding of external fixator performance.

The analysis incorporated two principal components: the tibial bone and the external fixator, following strategies employed in previous FEM‐based investigations of bone‐implant mechanics work of Blažević et al. [98], Ramlee et al. [99], and Wahab et al. [100]. The tibial bone presents a particular challenge to computational modelling because of its hierarchical microstructure [101], anisotropy [102], and heterogeneity [103], as well as its nonlinear [104], elastic‐plastic [105], and viscoelastic [106] properties under physiological loading. Cortical bone is capable of sustaining elastic deformation [107] followed by plastic yielding [108] and progressive microdamage [109], while trabecular bone exhibits pronounced nonlinear behaviour [110] due to its porous morphology [111]. Several advanced constitutive models have been proposed to capture these phenomena, ranging from elastic‐plastic laws (for example, Kelly and McGarry [112], O'Connor et al. [113], and Novitskaya et al. [114]) to viscoelastic formulations (such as Pawlikowski and Barcz [115], Gersie et al. [116], and Turek et al. [117]). However, in this study, the tibia was represented using a linear elastic, homogeneous, and time‐independent assumption. This simplification was chosen deliberately to prioritise computational efficiency and to ensure comparability between the ASTM F1541‐02 and tibia‐based frameworks. Furthermore, nation‐specific bone material data for Indonesian populations are currently lacking, necessitating the use of values derived from established literature. Although linear elastic models underestimate localised yielding [118], post‐yield plasticity [119], and microdamage phenomena [120]. They are widely recognised as sufficient for capturing first‐order stress distribution patterns and for comparative analyses of fixation devices.

Interactions among fixator components and between the fixator and tibia were modelled using bonded contact conditions, thereby assuming perfect continuity at all interfaces (threaded bar‐holder, bolt‐nut, and pin‐bone). This choice eliminated potential convergence instabilities and provided a consistent baseline for comparison across both test frameworks. In reality, these interfaces would experience micromotion, frictional sliding, and stress concentrations influenced by surgical technique [121], implant tolerances [122], and bone quality [123]. However, incorporating such complex behaviours would require extensive parameterisation of friction coefficients and surface conditions that were unavailable for the present study. Comparable bonded‐contact simplifications have been employed in previous FEM studies of orthopaedic fixation, as done by Shahar and Shani [124], Huang et al. [125], and Padovec et al. [126], where the emphasis was placed on structural rather than interface‐level mechanics.

The models were discretised using the automatic tetrahedral‐dominant meshing algorithm in ANSYS, which is particularly suited for representing complex geometries such as the irregular tibial surface and the intricate fixator assembly. An adaptive h‐refinement strategy [127] was applied to ensure solution accuracy without excessive computational cost. Regions of expected stress concentration, including the pin‐bone interface and bolt‐nut junctions, were assigned finer element sizes to enhance local resolution. On average, the element size was approximately 0.01 mm in critical regions and coarser in low‐stress areas, providing an efficient balance of accuracy and computational effort. The ASTM F1541‐02 configuration yielded approximately 800 finite elements and 3000 nodes, whereas the tibia‐based configuration consisted of 1000 finite elements and 3500 nodes. This discretisation level was selected based on a mesh sensitivity assessment using h‐refinement, which confirmed that further mesh refinement did not significantly alter the von Mises stress distributions in the regions of interest.

## 3 Results

**FIGURE 4:**
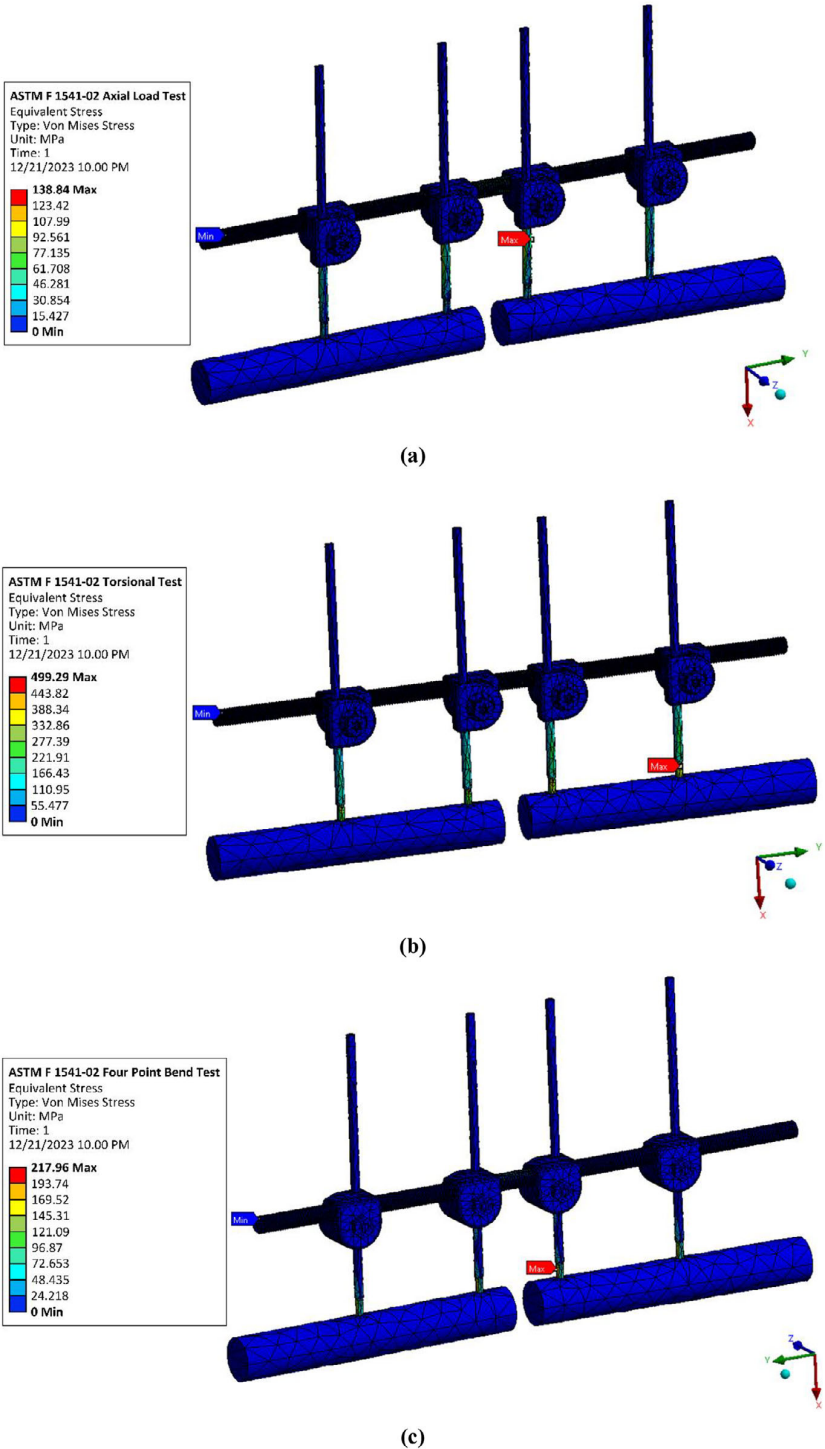
Von Mises stress contour of external fixator for ASTM F1541‐02 model: (a) Axial load test, (b) Torsional test and (c) Four‐point bend test.

**FIGURE 5:**
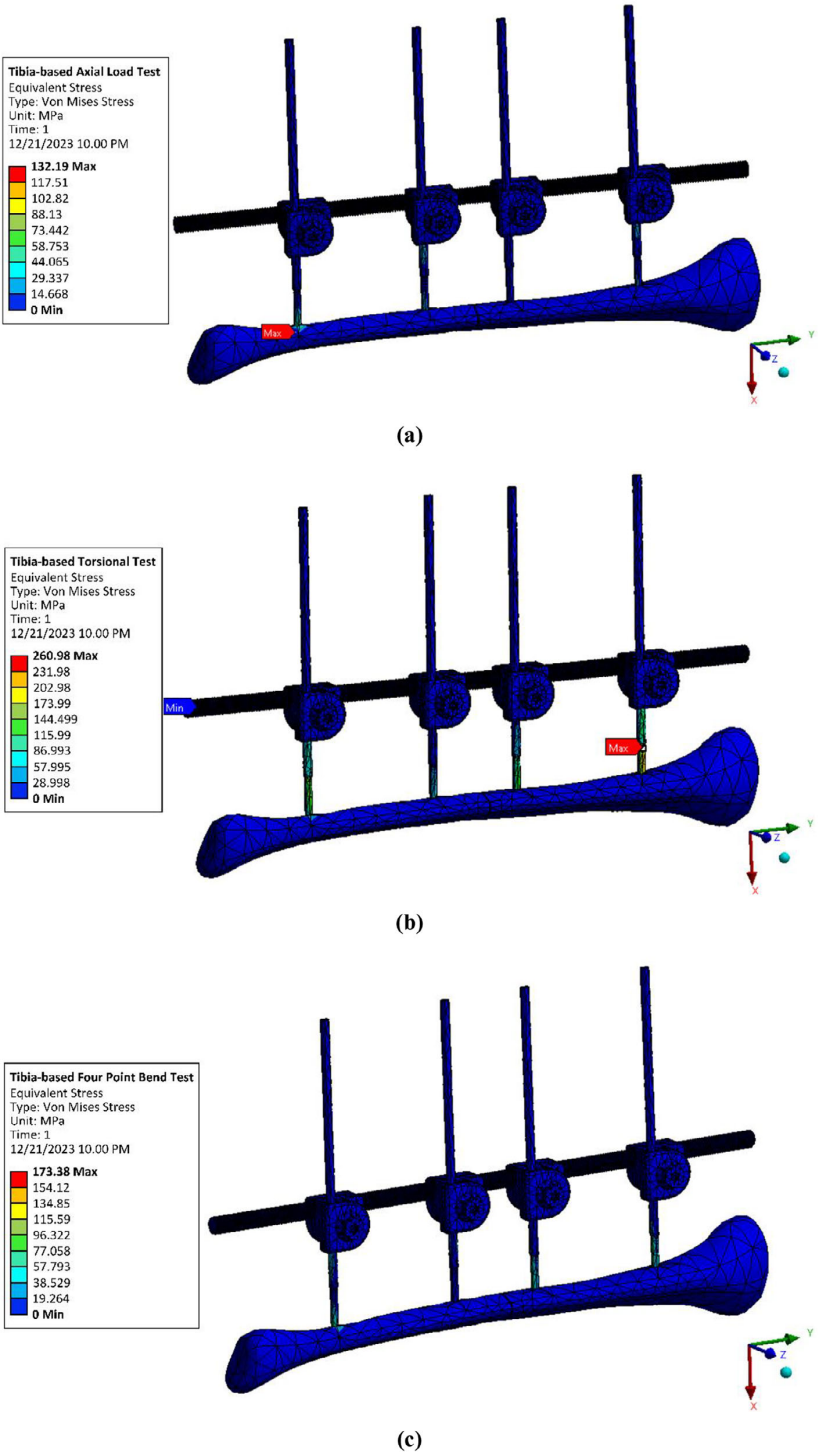
Von Mises stress contour of external fixator for tibia‐based model: (a) Axial load test, (b) Torsional test and (c) Four‐point bend test.

The distribution contours of von Mises stress in the external fixator obtained from the ASTM F1541‐02 framework are shown in Figure 4, while those from the tibia‐based framework are presented in Figure 5. Across both approaches, the stress distribution patterns revealed localized concentrations at regions of mechanical constraint, particularly around the pin–bone interfaces and bolt–nut connections, consistent with stress risers reported in previous studies of external fixation systems [96]. Among the three loading conditions, torsional loading produced the highest stress values, reaching 499.29 MPa in the ASTM F1541‐02 model and 260.98 MPa in the tibia‐based model. This outcome can be attributed to the relatively greater moment arm and rotational demand imposed on the fixator under torsional conditions, exacerbated by the spacing configuration of the pins. In particular, the placement of the second pin at a distance not optimised with respect to the first pin, as highlighted by prior recommendations [96], appears to contribute to increased torsional stress concentrations. These findings emphasise the sensitivity of external fixator mechanics to pin arrangement and loading conditions.

From a safety perspective, the maximum von Mises stresses in all simulated conditions remained below the yield strength of the materials considered. According to failure theory, a fixation construct can be regarded as safe if the maximum von Mises stress does not exceed the material's yield strength [128]. Both the ASTM F1541‐02 and tibia‐based models satisfied this criterion across axial, torsional, and four‐point bending loads, thereby indicating that the studied external fixator design possesses adequate safety margins for clinical use in Indonesian patients with open tibial fractures. These findings are particularly encouraging, as they suggest that the fixator can withstand physiologically relevant loading without risk of immediate structural failure, even when simplified assumptions regarding bone material behaviour and interface conditions are applied.

Quantitative comparisons between the two modelling frameworks are summarised in Tables 4 and 5. For axial loading, the maximum von Mises stresses were 138.84 MPa (ASTM F1541‐02) and 132.19 MPa (tibia‐based), reflecting a relatively small discrepancy of less than 5%. In contrast, torsional and four‐point bending conditions revealed substantially larger differences, with the ASTM framework predicting stresses more than 20% higher than those obtained from the tibia‐based model. These findings highlight a critical methodological consideration: while the ASTM F1541‐02 framework appears to approximate tibia‐based responses adequately under axial compression, it overestimates stresses under torsional and bending conditions. Consequently, reliance solely on ASTM‐based testing may lead to misinterpretation of fixator performance in loading scenarios involving complex rotations and bending, whereas tibia‐specific frameworks provide more physiologically realistic predictions.

**TABLE 4:** The maximum von Mises stress from ASTM F1541‐02 model.

Test type	Maximum von Mises Stress (MPa)
Axial load test	138.84
Torsional test	499.29
Four‐point bend test	217.96

**TABLE 5:** The maximum von Mises stress from tibia‐based model.

Test type	Maximum von Mises stress (MPa)
Axial load test	132.19
Torsional test	260.98
Four‐point bend test	173.38

## 4 Discussions

### 4.1 Implications of the Present Study

The findings of this study carry important implications for both clinical fracture management and the future development of external fixation systems. From a clinical standpoint, the results show the sensitivity of stress distribution to pin placement, fixator configuration, and loading conditions, particularly in torsional scenarios where the highest stress values were observed. Improper pin spacing or suboptimal alignment can exacerbate local stress concentrations, thereby reducing the long‐term stability of the construct and increasing the risk of complications such as pin loosening, mechanical failure [129], or delayed union [130]. These insights reinforce the need for surgical protocols that emphasize careful positioning of pins and rods to achieve optimal construct stiffness while minimizing torsional stress. Incorporating such biomechanical understanding into clinical decision‐making has the potential to improve outcomes for patients with open tibial fractures, particularly in resource‐constrained healthcare systems where revision surgeries are costly and burdensome.

For researchers and device developers, the results highlight the importance of computational models that integrate patient‐specific geometry [131]. While the ASTM F1541‐02 framework provides standardized testing procedures that are useful for regulatory compliance, its predictive reliability appears limited under non‐axial loading conditions such as torsion and bending. In contrast, tibia‐based models offer greater fidelity in capturing clinically relevant mechanical environments, thereby providing more accurate insights into device performance. This supports a shift towards incorporating patient‐specific anatomical datasets in both preclinical evaluation and device certification processes, aligning computational biomechanics more closely with the principles of personalised medicine [132]. Such integration could ultimately reduce the translational gap between in silico predictions and in vivo outcomes.

The demonstrated safety margins across all tested loading conditions also provide confidence in the viability of the external fixator design investigated in this study. However, the existence of substantial stress differences between ASTM‐based and tibia‐based simulations indicates that over‐reliance on standardised testing alone may lead to misinterpretation of device performance. For industry stakeholders, this finding explains the need to adopt dual validation strategies that combine standardized frameworks with anatomically representative models. Doing so could improve the robustness of design optimisation pipelines while ensuring that final products are not only mechanically sound but also tailored to the specific anatomical and clinical context of target populations, such as Indonesian patients.

More broadly, the present study emphasises the necessity of developing external fixators that balance safety, affordability, and accessibility. Through demonstrating that locally available biomaterials such as SS 304, AA 6061, Ti6Al4V, and SS 316L can meet safety thresholds under simulated physiological loading, this research provides a foundation for domestic production of external fixation devices in Indonesia. Reducing dependency on imported medical devices could alleviate national healthcare costs, while enabling local manufacturers to produce affordable and context‐specific solutions. This aligns with broader global health initiatives aimed at strengthening local biomedical industries in low‐ and middle‐income countries, thereby improving patient care and system sustainability.

### 4.2 Future Development of External Fixators

Building on the findings of the present study, several directions emerge for the future development of external fixators aimed at reducing postoperative complications and enhancing long‐term mechanical safety. A central focus should be placed on design optimization, particularly with respect to geometric configurations are pin spacing [133], rod positioning [134], and frame‐to‐bone distance [135]. Previous studies have demonstrated that these variables exert a significant influence on construct stiffness, load transfer, and overall biomechanical stability. Optimizing pin placement and rod alignment can reduce stress concentrations in both the fixator and the underlying bone, thereby minimizing the risk of mechanical failure and improving the clinical longevity of the device.

Another promising avenue involves the exploration of advanced biomaterials with improved strength‐to‐weight ratios [136], corrosion resistance [137], and biocompatibility [138]. While traditional metals such as stainless steel, aluminium alloys, and titanium alloys remain widely used in clinical settings [139] due to their accessibility [140] and proven performance [141]. Emerging materials, including bioresorbable composites [142], porous titanium scaffolds [143], and surface‐coated alloys [144] hold potential for enhancing both the mechanical and biological integration of external fixators. The application of 3D printing technologies could further facilitate patient‐specific device customisation [145], allowing frame geometries and material properties to be tailored to the anatomical and clinical requirements of individual patients. This trend aligns with the broader movement toward personalized medicine [146], in which treatment strategies are adapted to the specific biomechanical and physiological context of each patient.

In addition to material and design innovations, significant opportunities exist in the refinement of computational and experimental methodologies. Advances in high‐performance computing now allow for large‐scale parametric modelling [147] and multi‐objective optimisation [148], enabling the identification of optimal design trade‐offs between strength, stiffness, and weight. These improvements could be complemented by experimental validation through in vitro biomechanical testing [149], which remains essential for bridging the gap between numerical predictions and clinical outcomes. Establishing standardised protocols that combine ASTM‐based testing with tibia‐specific simulations could provide a comprehensive framework for device validation that satisfies both regulatory requirements and patient‐specific needs.

Equally important is the consideration of the surgical technique during device installation. Variability in intraoperative decisions, such as pin alignment, depth of penetration, and fixator‐to‐bone distance, can significantly influence postoperative construct durability and stress distribution [150]. Training protocols that integrate computational simulations with surgical planning tools could assist surgeons in anticipating biomechanical consequences and optimising fixation strategies before clinical application [151]. Such integration of computational biomechanics into clinical workflows could enhance decision‐making [152], reduce intraoperative variability [153], and ultimately improve patient outcomes [154].

### 4.3 Limitations of the Present Study

While the present study provides valuable insights into the biomechanical behaviour of external fixators through finite element analysis, several limitations must be acknowledged to contextualize the findings and outline avenues for future improvement. These limitations arise from simplifications in loading conditions, material modelling, fracture representation, geometric assumptions, and computational strategies, each of which may influence the applicability of the results to clinical practice.

First, the loading conditions applied in the simulations were restricted to a static compressive load of 500 N, representing an approximation of body weight during quiet standing. In reality, external fixators in clinical use are subjected to highly variable dynamic loading conditions [155] associated with patient activities such as walking [156], climbing stairs [157], or stumbling [158], which generate cyclic, impact, and multidirectional forces. The neglect of these dynamic aspects limits the ability of the present model to capture fatigue‐related phenomena [159], stress fluctuations [160], or the progressive loosening [161] that may occur under repetitive loading. Incorporating dynamic and patient‐specific loading scenarios into future computational analyses would enhance physiological realism and predictive accuracy.

Second, the frictional interactions between fixator components and the tibial bone were neglected, with all contacts modelled as frictionless or bonded. While this assumption ensured computational stability and facilitated direct comparison between the ASTM F1541‐02 and tibia‐based frameworks, it does not reflect clinical reality. In practice, bone–pin and component–component interfaces experience micromotion [162], slip [163], and variable friction coefficients that evolve over time [164], all of which can significantly influence load transfer [165] and fixation stability [166]. Similarly, the possibility of implant loosening under high stresses was not explicitly considered, despite being a clinically recognised complication of external fixation [167]. Future studies should therefore incorporate frictional contact definitions with experimentally validated coefficients, as well as interface debonding models, to more faithfully represent clinical fixation mechanics.

Third, the material modelling of the tibia represents a substantial simplification. Cortical and trabecular tissues exhibit anisotropic, heterogeneous, nonlinear, and time‐dependent behaviour [168], yet the present study adopted a homogeneous, isotropic, linear elastic representation. This decision was made to reduce computational cost and to maintain focus on the comparative evaluation of ASTM‐based and tibia‐based frameworks. However, the absence of elastic–plastic or damage parameters restricts the model's ability to predict post‐yield deformation [169], microcracking [170], or time‐dependent creep [171], which may occur under physiological loads. Incorporating advanced constitutive laws, such as elastic–plastic [172], viscoelastic [173], or damage‐based formulations [174], would enable more physiologically realistic predictions in future work.

Fourth, the fracture morphology was omitted from the tibial model, which was represented as a continuous structure without fracture gaps or callus tissue. This approach ensured controlled comparison of stress distributions within the fixator, but it does not reflect the clinical situation, where fracture type and callus development critically influence fixation mechanics and healing. The absence of explicit fracture geometry prevents assessment of how the external fixator interacts with evolving bone–callus structures during healing [175]. Future studies should therefore incorporate fracture‐specific models and staged callus development to improve physiological fidelity.

Fifth, the external fixator configuration was held constant based on a commercially available device. While this ensured comparability across frameworks, it restricted the exploration of design space and may limit generalisation to alternative fixator configurations. Parametric studies systematically varying these factors in patient‐specific models as conducted by Alqahtani [176], Li et al. [177], and Yang et al. [178] would provide more comprehensive guidelines for device optimization and clinical practice.

Sixth, although the tibial geometry was reconstructed from CT data, further validation of segmentation accuracy against cadaveric specimens would strengthen anatomical reliability [179]. Additionally, because nation‐specific bone property data for Indonesian populations were unavailable, the material values applied were sourced from global literature [85]. While widely used, these generic properties may not fully reflect local population‐specific bone characteristics, which could influence simulation outcomes [180]. Establishing an Indonesian‐specific database of bone material properties through experimental studies would improve the accuracy of future computational analyses [181].

Finally, limitations were also present in the numerical discretization strategy. The use of unstructured tetrahedral elements, while effective for modelling complex geometries [182], may lead to reduced accuracy in bending‐dominated conditions compared with structured hexahedral meshes [183]. Although mesh refinement was applied in critical regions and a sensitivity study ensured convergence of stress distributions, hybrid meshing strategies [184] and systematic mesh convergence [185] protocols could further improve numerical robustness in future work.

## 5 Conclusions

The present study established a finite element investigation of external fixators using two distinct frameworks: the standardized ASTM F1541‐02 method and a tibia‐based method reflecting patient‐specific anatomy. Through simulation of axial compression, torsional loading, and four‐point bending, areas of potential critical stress were identified, thereby providing new insight into the biomechanical behaviour of external fixators in the treatment of open tibial fractures in Indonesian patients. The results demonstrated that the maximum von Mises stresses generated in all loading scenarios remained below the yield strength of the evaluated biomaterials (SS 304, AA 6061, Ti6Al4V, and SS 316L). This finding indicates that the investigated external fixator design is mechanically safe under physiologically relevant conditions and can provide adequate structural support during clinical use. Importantly, the comparative framework revealed that the ASTM F1541‐02 approach yielded stress predictions consistent with tibia‐based results for axial loading, but produced significantly higher stress values in torsional and bending scenarios. These discrepancies suggest that while ASTM F1541‐02 remains useful for standardized axial testing, tibia‐specific models provide more reliable and physiologically realistic evaluations under complex non‐axial loading conditions. Beyond confirming the safety of the studied device, the findings carry broader implications for both clinical and engineering practice. For clinicians, the study emphasises the importance of pin placement and fixator configuration in minimising torsional stresses and ensuring long‐term construct stability. For researchers and manufacturers, the integration of tibia‐specific geometry into computational evaluation frameworks offers a more reliable pathway for device optimisation and regulatory assessment, particularly in populations with anatomical characteristics distinct from Western references.

## Author Contributions

The authors have significantly contributed to the development and the writing of this article. **Muhammad Kozin**: formal analysis, funding acquisition and writing – original draft. **Muhammad Imam Ammarullah**: formal analysis, investigation and writing – original draft. **Abdulfatah Abdu Yusuf**: project administration, validation, writing – review and editing. **Aghni Ulma Saudi**: data curation, formal analysis, writing – review and editing. **Siti Amalina Azahra**: software, visualisation, writing – review and editing. **I Nyoman Jujur**: data curation, visualisation, and writing – review and editing. **Muhammad Hirzan Arrifqi**: conceptualisation, methodology, and writing – review and editing. **Moch. Agus Choiron**: resources, supervision, and writing – review and editing.

## Declaration of AI use

The authors declare the use of generative artificial intelligence (AI) and AI‐assisted technologies in the preparation of this manuscript. Specifically, ChatGPT (version GPT‐5, OpenAI) was used to improve the readability and language of the article. The AI tool was not used for data analysis, interpretation of results, or drawing scientific conclusions. All content has been reviewed and verified by the authors to ensure accuracy and integrity.

## Funding

This work was funded by the Riset dan Inovasi untuk Indonesia Maju (RIIM) Batch 1 Program under contract numbers 65/II.7/HK/2022 and 27/II.7/HK/2023.

## Declaration of Interest's Statement

One of the authors, Muhammad Hirzan Arrifqi, is employed by PT. Zenith Allmart Precisindo, Indonesia. However, the company had no role in the design of the study, data collection, computational modeling, analysis, interpretation of results, or the preparation of this manuscript. The research was conducted independently, and no financial or commercial influence was exerted by the company.

## Ethics Statement

This study did not involve human participants or animals, and no ethical approval was required. All research procedures adhered to relevant ethical guidelines and best practices for non‐human and non‐animal research.

## Consent

The authors consent for the publication of this manuscript.

## Conflicts of Interest

The authors declare no conflicts of interest.

## Transparency Statement

The authors affirm that this manuscript is an honest, accurate, and transparent account of the study being reported; that no important aspects of the study have been omitted; and that any discrepancies from the study as planned (and, if relevant, registered) have been explained.

## Data Availability

All data generated or analysed during this study are included in this published article. No additional datasets were generated or analysed beyond the contents of the manuscript.
